# Supplementary material for: Graphia: A platform for the graph-based visualisation and analysis of high dimensional data
Source: PLoS Comput Biol. 2022 Jul 25;18(7):e1010310. doi: 10.1371/journal.pcbi.1010310 (PMC9352203; doi:10.1371/journal.pcbi.1010310)
Supplement: S1 Fig — Three graphs were used for these tests, a correlation graph (panels a, d, g) generated from the GNF mouse gene expression atlas using Graphia (r = 0.7), then saved as a.gml file, and two graphs from an online repository https://chriswalshaw.co.uk/partition/#graphs - finan512 (panels b, e, h) and fe_pwt (panels c, f, i). These were selected to represent large graphs of different node/edge counts and structure. Each graph was opened using the three tools and the time taken to load and layout the graph shown in the panel recorded. In each case, a force directed layout algorithm similar to that employed by Graphia was used; for Gephi (v0.9.2) this was the Force Atlas 2 layout algorithm, and for Cytoscape (v3.9.0) the OpenCL Prefuse Layout algorithm. In different views shown, we have not attempted to fully optimise graph layout in each case but show the layouts at 10x the standard number of iterations for Gephi and Cytoscape. While these layouts completed relatively quickly, they were far from ‘finished’ in establishing a stable layout. By contrast, we ran Graphia’s (v3.0) dynamic layout to a point where layout was near optimal which took considerably longer. A notable difference between the network tools was in speed and fluidity of interaction with the graph visualisation, i.e. fps (frames per second) rates, Graphia being an estimated 3–4 times quicker than within Gephi or Cytoscape and in 3D. The specifications of the computer used for these tests are: Intel Core i7-4930K, 16Gb memory, Nvidia GeForce RTX 2060 Super, Windows 10 Pro (10.0.19043). (DOCX) [file pcbi.1010310.s001.docx]

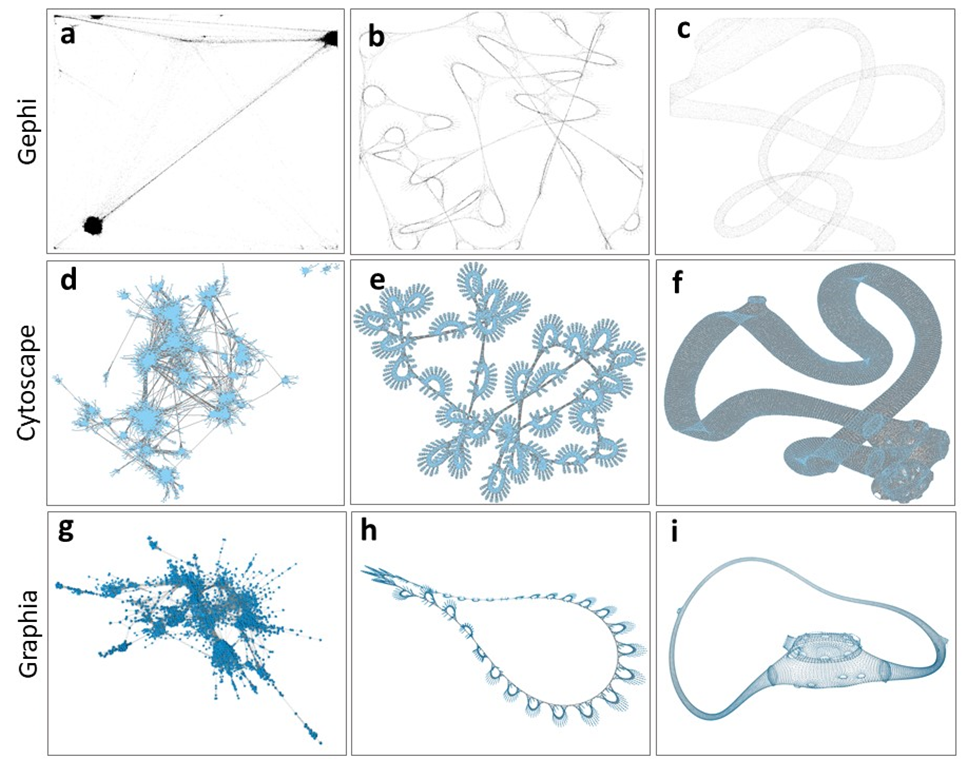


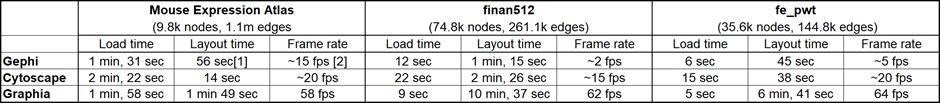


S1 Fig. Comparison of Gephi, Cytoscape and Graphia in terms of large graph loading, layout and rendering performance. Three graphs were used for these tests, a correlation graph (panels a, d, g) generated from the GNF mouse gene expression atlas using Graphia (r = 0.7), then saved as a .gml file, and two graphs from an online repository https://chriswalshaw.co.uk/partition/#graphs - finan512 (panels b, e, h) and fe_pwt (panels c, f, i). These were selected to represent large graphs of different node/edge counts and structure. Each graph was opened using the three tools and the time taken to load and layout the graph shown in the panel recorded. In each case, a force directed layout algorithm similar to that employed by Graphia was used; for Gephi (v0.9.2) this was the Force Atlas 2 layout algorithm, and for Cytoscape (v3.9.0) the OpenCL Prefuse Layout algorithm. In different views shown, we have not attempted to fully optimise graph layout in each case but show the layouts at 10x the standard number of iterations for Gephi and Cytoscape. While these layouts completed relatively quickly, they were far from ‘finished’ in establishing a stable layout. By contrast, we ran Graphia’s (v3.0) dynamic layout to a point where layout was near optimal which took considerably longer. A notable difference between the network tools was in speed and fluidity of interaction with the graph visualisation, i.e. fps (frames per second) rates, Graphia being an estimated 3-4 times quicker than within Gephi or Cytoscape and in 3D. The specifications of the computer used for these tests are: Intel Core i7-4930K, 16Gb memory, Nvidia GeForce RTX 2060 Super, Windows 10 Pro (10.0.19043).
